# Adherence to treatment to help quit smoking: effects of task performance and coping with withdrawal symptoms

**DOI:** 10.1186/1471-2458-14-1217

**Published:** 2014-11-25

**Authors:** Francisca López-Torrecillas, Maria Mar Rueda, Eva María López-Quirantes, Javier Machado Santiago, Reyes Rodríguez Tapioles

**Affiliations:** Departamento de Personalidad, Evaluación y Tratamiento Psicológico, Centro de Investigación Cuerpo Cerebro Comportamiento (CIMCYC), Universidad de Granada, Campus Universitario de Cartuja s/n, 18071 Granada, España; Departamento de Estadística e Investigación Operativa, Universidad de Granada, Granada, España; Área de Medicina del Trabajo (Servicio de Prevención), Universidad de Granada, Granada, España

**Keywords:** Treatment to quit smoking, Treatment adherence

## Abstract

**Background:**

Currently the combined cognitive-behavioral and pharmacological treatment is the best option to quit smoking, although success rates remain moderate. This study aimed to identify predictors of continuous abstinence in an assisted smoking cessation program using combined treatment. In particular, we analyzed the effects of socio-demographic, smoking-, and treatment-related variables. In addition, we analyzed the effect of several risk factors on abstinence, and estimated a model of risk for smoking relapse.

**Methods:**

Participants were 125 workers at the University of Granada (50 males), with an average age of 46.91 years (SD = 8.15). They were recruited between 2009 and 2013 at an occupational health clinic providing smoking cessation treatment. Baseline measures included socio-demographic data, preferred brand of cigarettes, number of years smoking, use of alcohol and/or tranquilizers, past attempts to quit, Fargerström Test for Nicotine Dependence, Smoking Processes of Change Scale, and Coping with Withdrawal Symptoms Interview. Participants were invited to a face-to-face assessment of smoking abstinence using self-report and cooximetry hemoglobin measures at 3, 6, and 12 months follow-up. The main outcome was smoking status coded as “relapse” versus “abstinence” at each follow-up. Kaplan-Meier survival analysis was performed to estimate the probability of continued abstinence during 12 months and log-rank tests were used to analyze differences in continued abstinence as a function of socio-demographic, smoking-, and treatment-related variables. Cox regression was used to analyze the simultaneous effect of several risk factors on abstinence.

**Results:**

Using alcohol and/or tranquilizers was related to shorter abstinence. Physical exercise, the number of treatment sessions, performance of treatment tasks, and coping with withdrawal symptoms were related to prolonged abstinence. In particular, failure to perform the treatment tasks tripled the risk of relapse, while lack of coping doubled it.

**Conclusions:**

Our results show that physical exercise, performance of treatment-related tasks, and effective coping with withdrawal symptoms can prolong abstinence from smoking. Programs designed to help quit smoking can benefit from the inclusion of these factors.

## Background

Tobacco consumption remains the number one preventable cause of morbidity and mortality, responsible for 31% of lung cancer cases. Other types of cancer associated with tobacco consumption are lip, mouth, pharynx, larynx, esophagus, bladder, and kidney cancer. Further, exposure to environmental (i.e., second hand) tobacco smoke increases the risk of lung cancer, as well as the risk of suffering respiratory, cardiovascular, and other chronic diseases [1]. Despite several decades of falling smoking prevalence rates, the National Spanish Health Survey [2] now shows that this decline has stalled, with 27.9% of men and 20.2% of women over age 16 smoking daily. Currently the combined cognitive-behavioral and pharmacological treatment is the best option to quit smoking [3–8]. General population surveys show that about 70% of smokers would like to quit and about 30-40% try to do so, but the proportion of those who succeed is less than 5% [9–11]. Recent studies have identified a number of variables associated with success at quitting smoking [12–19]. Among these are socio-demographic variables such as age, gender, education level, and occupation. To illustrate, younger men (aged 22-38 years) are more likely to continue smoking than older men [18] and women are less likely to quit than men [13]; education level and occupation have not been associated with quitting success [16].

Other factors investigated extensively are the severity of tobacco dependence and its relation to treatment outcomes. In particular, the number of cigarettes, the intensity of tobacco dependence, and alcohol consumption decrease the probability of quitting [15–17]. Treatment-related variables can also influence quitting success. For example, past attempts to quit predicted abstinence during the first week and sustained abstinence during 6 months in a second attempt to quit [15]. Another factor that can influence treatment adherence is the number of treatment sessions [4, 19]. For example, having completed more than four treatment sessions and performed treatment tasks predicted abstinence at 6 months [17]. Finally, the motivation to change has also been related to quitting success [20].

Treatment adherence is defined as the degree to which the person's behavior follows health recommendations. It includes the patient's ability to attend scheduled appointments, take medications as indicated, make the recommended changes in lifestyle, and complete the laboratory studies or tests requested [21]. There are several methods to measure treatment adherence and each of them has some limitations. Hence, several methods should be used simultaneously in order to gather as much information as possible. For instance, it has been proposed to use three variables to assess adherence: the completion of tasks assigned during treatment, the number of completed treatment sessions, and the extent of coping with withdrawal symptoms [22]. Finally, some studies emphasize the role of physical exercise in coping with craving and withdrawal symptoms (depressed mood, negative affect, insomnia, stress, and weight gain) [23–25]. For instance, performing moderate exercise reduces craving and this effect is maintained for more than 20 minutes after exercise completion [26, 27].

The objective of this study was to identify predictors of continuous abstinence during 12 months in an assisted smoking cessation program. In particular, we investigated the influence of socio-demographic variables (age, gender, education level, and occupation), smoking-related variables (cigarette brand, nicotine concentration mg per cigarette, number of cigarettes smoked daily, nicotine dependence, years of smoking, use of alcohol and/or tranquilizers) and treatment-related variables (past attempts to quit, compliance with the pharmacological therapy, performance of treatment tasks, number of treatment sessions, coping with withdrawal symptoms, and physical exercise). Finally, we analyzed the simultaneous effect of several risk factors on abstinence and estimated a model of risk for smoking relapse.

## Methods

### Participants

Participants were 125 workers at the University of Granada (50 men and 75 women) with a mean age of 46.91 years (SD = 8.15). They were recruited between 2009 and 2013 at an occupational health clinic providing smoking cessation treatment. Participants smoked a mean number of 19.86 (SD = 8.95) cigarettes daily. The mean score on the Fagerström test for nicotine dependence was 4.62 (SD = 2.24). Participants entered the study if they were 18 years of age or older, had an employment contract with the University of Granada, wanted to voluntarily participate in the treatment, and correctly filled in the pre-treatment evaluation measures. Participants were excluded if they were diagnosed with a serious mental disorder (bipolar and/or psychotic disorder, etc.), had a concurrent dependence on other substances (cocaine, heroin, alcohol, etc.), or were regularly taking medications that were incompatible with the pharmacological treatment used in the therapy. Participants were assessed at baseline with the measures outlined below. As part of the treatment patients underwent cognitive-behavioral therapy, which included the performance of tasks at home, and pharmacological therapy with varenicline. Participants were informed about the aims of the study and provided signed informed consent. Ethical approval was obtained from the Ethics Committee, Research University of Granada, Spain.

### Procedure

An initial evaluation of the smokers was performed in a single session at the beginning of the program, in which the instruments described below were administered. The evaluation, treatment, and 3-, 6- and 12-month follow-up sessions of the program were implemented individually.

The program consisted of three phases. The first phase included a personalized number of sessions until abstinence was reached. During the first phase the cognitive-behavioral therapy was initiated by working towards developing stimulus control and self-control, and reducing tobacco consumption. Once the smoker had reduced tobacco consumption by 80%, pharmacological treatment with varenicline was started. We chose this scheme for two reasons. First, this was a way to ensure that the participant was sufficiently motivated to receive the free pharmacological treatment and quit smoking. Second, this way the participant starts to develop his or her self-control and management of symptoms. Varenicline is a nicotinic receptor partial agonist that effectively aids smoking cessation. The treatment starts with a daily dose of 0.5 mg for three days, and increases to a dose of 1 mg twice a day until the end of the treatment. The duration of the treatment with varenicline was 12 weeks. Adherence was defined as accurately following the prescribed regimen. Prescription of varenicline was administered in line with the Food and Drug Administration’s guidelines [28, 29]. The cost of the medication was covered by the Prevention Service (Area Labor Medicine) of the University of Granada. If the participants did not reach abstinence, they abandoned the program. Participants who reached abstinence proceeded to the second phase of the program which consisted of 6 sessions and aimed to maintain abstinence. It included training in problem solving, coping, and behaviors alternative to smoking. Participants also performed tasks at home. These tasks included reporting their smoking behavior, cravings, coping, and problem solving. The third phase consisted of three follow-up sessions (at 3, 6, and 12 months). Participants were telephonically contacted by an independent assessor (blind to the study purpose and methods) at each endpoint (3, 6 and 12 months after the start of the program). The purpose was to monitor their compliance with the treatment and their willingness to participate in the follow-up face-to-face assessments of smoking abstinence. Abstinence was assessed with a self-report of smoking behavior which was cross-validated with measurement of patients’ co-oximetry hemoglobin levels. Participants’ outcomes were coded as “relapse” or “abstinence”. Abstinence was defined as not having smoked even once since the day the participant quit after gradual reduction of nicotine intake and pharmacological treatment. Relapse was defined as having smoked for 7 consecutive days in the past 3, 6 and 12 months, respectively [30–32].

### Instruments

*Semi-structured interview for smokers*[33]. This instrument provides information about socio-demographic data, preferred brand of cigarettes, number of years smoking, usage of other substances, and past attempts to quit.

*Fargerström Test for Nicotine Dependence*[34]. This instrument evaluates the intensity of physical addiction to nicotine and has a consistent factorial structure [35]. In this study we used the Spanish version of the test [36].

*Smoking Processes of Change Scale* (SPC) [37, 38]. This questionnaire measures 10 basic processes of change. Participants indicated the frequency with which they have engaged in or experienced 40 activities or events within the last month on a 5-point Likert scale from (1) never to (5) repeatedly. This instrument has good psychometric properties. In this research we used the Spanish version of the instrument [39].

*Coping with Withdrawal Symptoms Interview* (CWSI). This instrument was designed specifically for this research. Each item had 4 response alternatives, on a 4-point Likert scale from (1) never to (4) repeatedly. Smokers were asked about (1) craving in the past month, (2) coping with craving, (3) presence of anxiety, (4) depression, (5) sleeping problems, (6) eating problems (excess appetite), (7) physical problems like stomach pain as a result of drug therapy, or other physical problems, (8) whether they had done physical exercise in order to cope with withdrawal symptoms and (9) whether they perceived any benefits after quitting smoking. Exercise was measured following the criteria of the International Physical Activity Questionnaire (IPAQ) [40, 41]. In particular, participants answered 9 questions on a 5-point Likert scale about the intensity, frequency, and duration of physical activity they did in the past month. Based on their averaged responses to these questions, participants were classified in three groups: frequent exercise, moderate exercise, and inactive. Based on their responses on all interview questions, participants were classified into three coping groups: lack of coping and recognition of the problem, i.e., ineffective coping (score > 4), moderate coping (score 6 to 12), and effective coping (score 13 to 17).

### Statistical analysis

Discrete-time survival methods were used to analyze how the variation in risk of smoking relapse over time was related to the socio-demographic, smoking-, and treatment-related variables. The survival time of participants who did not smoke during the observation period was set to the end of the data collection window [42]. Event status was coded as 0 = relapse (smoked prior to termination time) or 1 = still abstinent at termination time. A predictor was retained in the model if it improved the overall goodness of fit of the model. The effects of the continuous predictors were displayed by plotting survival functions using Kaplan–Meier graphs [43] and estimating the median life-time, the time at which half the sample had experienced the event and half had not [44]. Last, Cox proportional hazard regression models were used to calculate the hazard rate ratios [14, 45, 46]. The survival analysis was conducted with the SPSS software package.

## Results

### Abstinence rates

At 1-month follow-up, 75 participants had maintained abstinence during treatment. Therefore, the abstinence rate was 60%. At 3-month follow-up, 70 participants had maintained abstinence during treatment (abstinence rate = 56%). At 6-month follow-up, 56 participants had maintained abstinence during treatment (abstinence rate = 44.8%). At the 12-month follow-up, 45 participants had maintained abstinence during treatment (abstinence rate = 36%) (see Table 1).

**Table 1:** **Number of participants classified as abstinent vs. in relapse**

Time	Abstinence	Relapse	% Abstinence rate
**1- month**	75	50	60%
**3 –month**	70	55	56%
**6- month**	56	69	44.8%
**12- month**	45	80	36%

Abstinence was defined as not having smoked even once since the day the participant quit after gradual reduction of nicotine intake and pharmacological treatment. Relapse was defined as having smoked for 7 consecutive days in the respective period.

### Socio-demographic variables

Survival analysis results (Table 2) revealed that the abstinence duration was not different across groups determined by socio-demographic characteristics (age, gender, education level, and occupation).

**Table 2:** **Survival analysis results: effects of socio-demographic variables**

Variables	Abstinence					95% IC	Log rank (Mantel-Cox)	*p*
	Sample size	Number	Rates	Mean	Standard error			
**Age**								
27-37	16	11	68.8%	6.73	1.54	3.71- 9.74	1.833	.400
38-48	54	41	75.9%	8.32	.73	6.89 - 9.75		
49-63	55	43	78.2%	7.07	.72	5.66 - 8.48		
**Gender**								
Male	50	39	78%	7.85	.77	6.34 - 9.35	.247	.619
Female	75	56	74.7%	7.38	.63	6.14 - 8.61		
**Education**								
Elementary school	63	47	74.6%	7.06	.69	5.70 - 8.42	2.255	.324
College degree	43	31	72.1%	8,65	.81	7.07- 10.22		
Ph.D.	19	17	89.5%	7,00	1.23	4.58 - 9.42		
**Occupation**								
Janitorial	18	15	83.3%	7.40	1.23	4.99 - 9.80	1.318	.517
Administrative and service personnel	80	58	72.5%	7.95	.62	6.72- 9.17		
Teachers and researchers	27	22	81.5%	6.68	1.03	4.66- 8.70		

### Smoking-related variables

**Figure 1:**
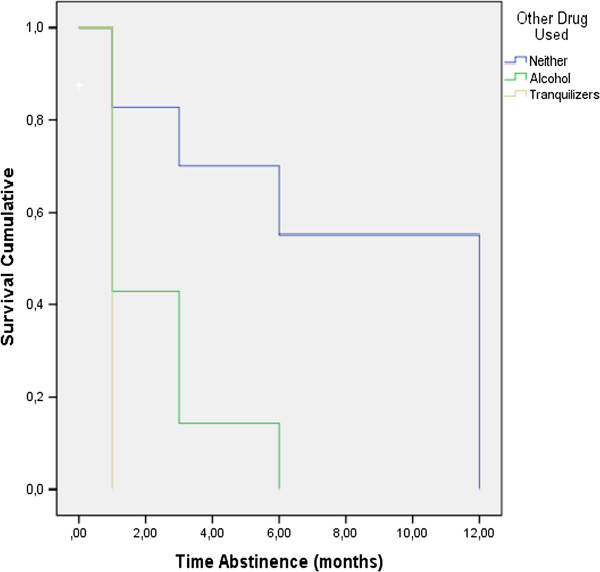
**Survival curve depending on the use of another substance.**

Survival analysis results (Table 3) showed that the abstinence duration for the different categories of the variable *use of alcohol and/or tranquillizers* differed significantly (Log Rank =17.679; p =0.001), i.e., using another substance had a significant influence on the probability to remain abstinent. Figure 1 shows the survival curve depending on the use of another substance. The group that used no other substance showed greater survival than the group with alcohol use; the group with alcohol use in turn showed greater survival than the group that used tranquilizers. The p-values in Table 3 indicate that there were no significant differences in abstinence duration with respect to the variables *cigarettes brand, nicotine concentration mg/cigarette, number of cigarettes smoked daily, score on the Fagerström test,* and *years of smoking*.

**Table 3:** **Survival analysis results: effects of smoking-related variables**

Variables	Abstinence					95% IC	Log rank (Mantel-Cox)	*p*
	Sample size	Number	Rates	Mean	Standard error			
**Cigarette brand**								
Blonde	105	80	85%	7.54	.54	6.48 - 8.59	3.931	.140
Black	12	9	75%	5.90	1.35	3.25 - 8.53		
Rolling	8	6	73.1%	10.50	1.50	7.56 - 13.44		
**Nicotine concentration mg per cigarette**								
>1	23	20	85%	6.45	1.10	4.29 - 8.61	1.619	.44
>1 > 1.5	99	72	75%	7.90	.56	6.82 - 8.99		
>1.5 2	3	3	73.1%	7.00	2.65	1.81 - 12.19		
**Number of cigarettes smoked daily**								
> 10	20	17	85%	7.94	1.14	5.71 - 10.17	.186	.980
>10 > 20	72	54	75%	7.44	.64	6.19 - 8.70		
>20 > 30	26	19	73.1%	7.58	1.13	5.37 - 9.78		
>30	7	5	71.4%	7.60	2.69	2.32 - 12.88		
**Fagerström test score**								
>6	99	77	77.8%	7.69	.54	6.63 - 8.75	.296	.586
>7	26	18	69.2%	7.06	1.14	4.83 - 9.28		
**Years of smoking**								
> 10 years	14	12	85.7%	7.17	1.47	4.28 - 10.05	2.560	.464
> 20 years	28	19	67.9%	8.26	1.09	6.12 - 10.40		
> 30 years	40	33	82.5%	8.21	.82	6.60 - 9.83		
> 30 years	43	31	72.1%	6.61	.84	4.98 - 8.25		
**Use of alcohol and/or tranquilizers**								
Neither	116	87	75%	8.07	.50	7.10 - 9.04	17.679	.001
Alcohol	7	7	100%	2.29	.71	.89 - 3.69		
Tranquilizers	2	1	50%	1.00	.00	1.00 - 1.00

### Treatment-related variables

**Figure 2:**
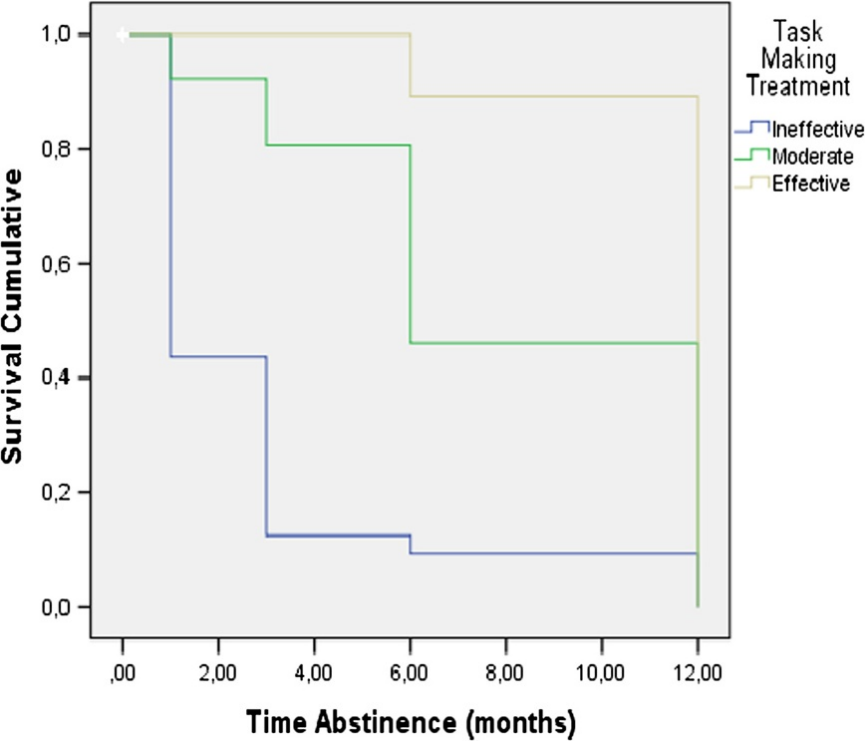
**Survival curve depending on performance of treatment tasks.**

**Figure 3:**
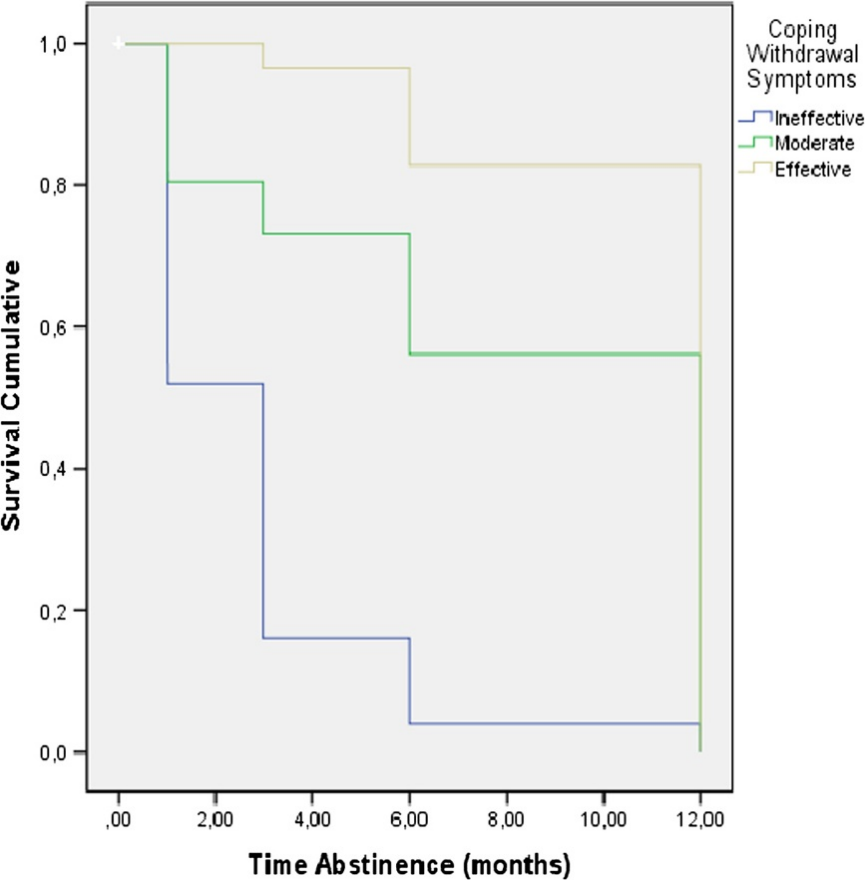
**Survival curve depending on coping with withdrawal symptoms.**

**Figure 4:**
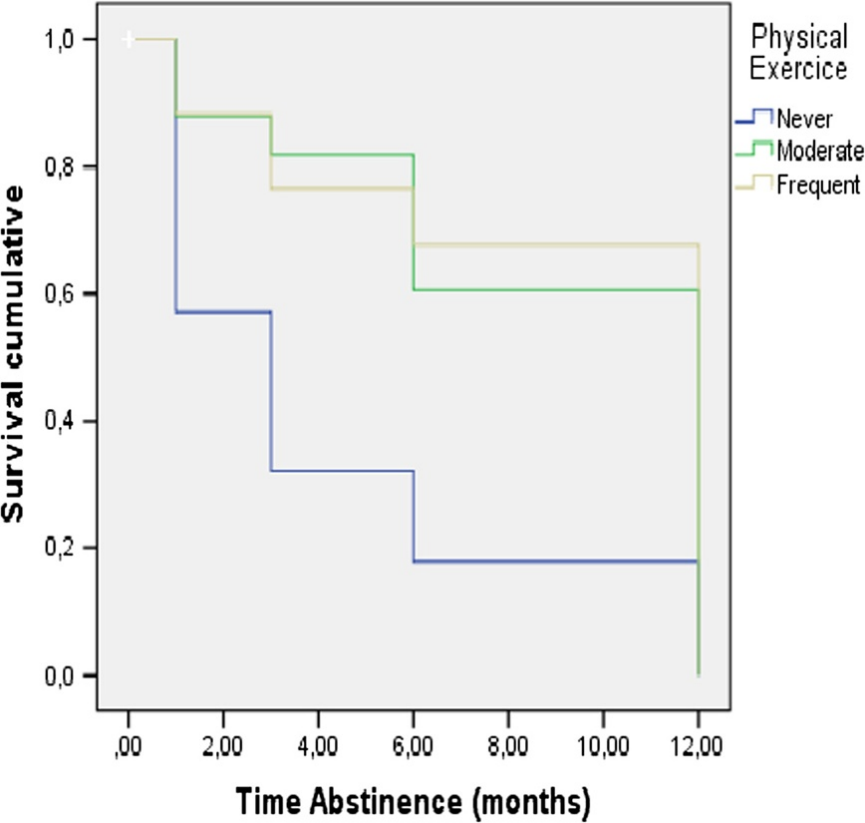
**Survival curve depending on physical exercise.**

Survival analysis results (Table 4) revealed that the following variables had a significant influence on survival time: *performance of treatment tasks,* Log Rank = 68.330, p = 0.001, *number of treatment sessions*, Log Rank = 8.283, p = 0.041, *coping with withdrawal symptoms*, Log Rank = 49.750, p = 0.000, and *physical exercise,* Log Rank = 23.376, p = 0.001. Figures 2, 3 and 4 illustrate the survival curves for abstinence duration for the different levels of these variables. In particular, Figure 2 shows that participants in the *effective task performance* and *moderate task performance* groups have a better survival curve than the *ineffective task performance* group. Table 4 shows that participants who completed *16-20 treatment sessions* had a better survival curve than those who completed *11-15, 6-10 or 1-5 treatment sessions.* Figure 3 shows that participants who were classified in the groups *effective coping* and *moderate coping* had a better survival curve than those classified into *ineffective coping.* Figure 4 shows that participants in the *frequent* and *moderate exercise* groups have a better survival curve than participants who were classified as *never engaging in physical exercise*. Past attempts to quit and *compliance with the pharmacological therapy* had no significant influence on abstinence duration.

**Table 4:** **Survival analysis results: effects of treatment-related variables**

Variables	Abstinence					95% IC	Log rank (Mantel-Cox)	*p*
	Sample size	Number	Rates	Mean	Standard error			
**Past attempts to quit (N)**								
0	31	19	96.3%	7.21	1.13	4.99 - 9.43	4.415	.220
>2	79	63	79.7%	7.79	.59	6.64 - 8.95		
>7	12	11	91.7%	7.91	1.51	4.95 - 10.87		
>7	3	2	91.7%	2.00	1.00	.04 - 3.96		
**Compliance with the pharmacological therapy**								
Not	33	8	24.24%	7.38	1.83	3.79 -10.97	.008	.931
Yes	92	87	94.56%	7.59	.507	6.59 - 8.58		
**Performance of treatment tasks**								
Not	61	32	52.5%	2.81	.57	1.69 - 3.93	68.330	.001
Moderate	26	26	100%	8.04	.79	6.50 - 9.58		
Yes	38	37	97.4%	11.35	.31	10.7 - 11.96		
**Number of treatment sessions**								
1-5	43	23	53.5%	6.78	1.03	4.77 – 8.80	8.283	.041
6-10	60	52	86.7%	8.71	.63	7.52 – 9.91		
11-15	14	12	85.7%	6.08	1.38	3.37 - 8.80		
16-20	8	8	100%	4.63	1.72	1.25 - 8.00		
**Coping with withdrawal symptoms**								
Ineffective	49	25	51%	2.76	.51	1.77 - 3.76	49.750	.001
Moderate	46	41	89.1%	8.17	.73	6.75 - 9.59		
Effective	30	29	96.7%	10.86	.48	9.92 - 11.80		
**Physical exercice**								
Never	49	28	57.1%	4.18	.77	2.67 - 5.69	23.376	.001
Moderate	42	33	78.6%	8.85	.74	7.41 - 10.29		
Frequent	34	34	100%	9.12	.75	7.65 - 10.59

### Cox regression analysis

We performed a Cox regression analysis to identify risk factors associated with the probability of survival. We fitted a model including all factors related to tobacco consumption and treatment which were significant in the survival analysis. We included two additional quantitative variables: processes of change and number of days it took for the smoker to achieve abstinence. Table 5 shows that the variables *performance of treatment tasks* and *coping with withdrawal symptoms* were significant (p = 0.01 and 0.013, respectively). The negative sign of the beta-coefficients (*B*) indicates the direction of the relationship; i.e., these variables are protective factors for abstinence from smoking (Hazard ratio >1). *Processes of change, days to achieve abstinence, other drug used, number of treatment sessions, and physical exercise* were not significant (*p* >0.05) in the regression model. Table 6 shows a comparison between the levels of the variable *performance of treatment tasks*. Specifically, the group which did not perform the tasks had 3 times higher risk of smoking relapse than the group performing tasks moderately [Hazard ratio; (H.R = 3.032)]. No differences in survival are observed between the group performing tasks moderately and the group performing tasks effectively. Also Table 6 shows a comparison between the levels of the variable *coping with withdrawal symptoms*. Specifically, the group which coped ineffectively had 2.4 times higher risk of smoking relapse than the group which coped moderately [Hazard ratio (H.R = 2.410)]. No differences in survival were observed between participants who coped moderately and effectively.

**Table 5:** **Results from Cox regression analysis on the probability to remain abstinent at 12 months follow-up**

Variables	B	*p*	H.R	95.0% IC
Motivation for change	-.018	.778	.982	.868 - 1.112
Days to achieve abstinence	-.004	.519	.996	.984 - 1.008
Use of alcohol and/or tranquilizers	.573	.107	1.773	.883 - 3.561
Number of treatment sessions	.042	.134	1.043	.987 - 1.103
Physical exercice	-.021	.889	.979	.725 - 1.322
Performance of treatment tasks	-.547	.001	.579	.422 - .794
Coping with withdrawal symptoms	-.070	.013	.933	.882 - .986

**Table 6:** **Comparison between the levels of the variables** ***performance of treatment tasks*** **and** ***coping with withdrawal symptoms*** **in Cox regressions**

Variables	B	*p*	H.R	95.0% IC
**Performance of treatment tasks**		.003		
Ineffective - Moderate	1.109	.001	3.032	1.601 - 5.743
Effective - Moderate	.321	.231	1.378	.815 - 2.330
**Coping with withdrawal symptoms**		.051		
Ineffective - Moderate	.880	.016	2.410	1.178 - 4.930
Effective - Moderate	.206	.409	1.229	.753 - 2.006

## Discussion and conclusions

The aim of this study was to identify predictors of continuous abstinence in an assisted smoking cessation program using combined cognitive-behavioral and pharmacological therapy. In particular, we investigated the influence of socio-demographic, smoking-, and treatment-related variables. We analyzed the effect of several risk factors on abstinence and modeled the probability of remaining abstinent at follow-up. In particular, we utilized baseline psychometric assessments and predicted smoking status at three endpoints: 3, 6 ,and 12 months after the start of the program.

Results from the survival analysis revealed that socio-demographic variables were not related to abstinence. These results are in line with some previous studies [15, 16]. However, other studies [5, 20] have found that socio-demographic variables, smoking habits, past attempts to quit, and motivation for change are associated with smoking relapse. These discrepancies may be due to the fact that smokers are a heterogeneous group [12].

We found that using alcohol and/or tranquilizers influenced abstinence. In particular, patients who did not use any substances had better survival curves than those who consumed alcohol. Further, patients who were using tranquilizers had an even worse survival curve than those who consumed alcohol. These results are in line with previous findings showing that alcohol and tranquilizers can be a coping response to nicotine withdrawal [14]. The brand of cigarettes, nicotine concentration mg/cigarette, number of cigarettes smoked daily, score on the Fagerström test, and years of smoking were unrelated to abstinence. This lack of effects is inconsistent with results from previous studies where nicotine dependence and motivation for change predicted quitting success and high scores on the Fageström test and the number of previous attempts to quit smoking predicted abstinence one week and 6 months post-treatment [12, 15, 19].

Most importantly, our results showed that performance of treatment tasks, the number of treatment sessions, coping with withdrawal symptoms, and physical exercise had a significant influence on the duration of abstinence. While the effect of number of treatment sessions has been established by previous research [4, 9, 19], this is the first study to our knowledge to link effectiveness of treatment task performance to continued abstinence one year after the start of treatment.

Specifically, participants who performed the treatment tasks effectively were abstinent for longer than participants who performed them less effectively. In particular, the group that did not perform the tasks had a 3 times larger risk of relapse. Our results suggests that giving “homework” to smokers in the form of regularly reporting on their smoking behavior, cravings, or coping behavior, should be implemented in programs designed to help quit smoking. Further research is needed to investigate what factors are related to performing such tasks effectively and how they should be implemented in order to maximize completion rates.

Both effective and moderately effective coping with withdrawal symptoms resulted in longer abstinence than coping ineffectively. In particular, the group of participants classified as coping ineffectively had a 2.4 times higher risk of relapse than the group that coped moderately*.* To our knowledge this is the first study to assess the effectiveness of coping with withdrawal symptoms and its impact on the duration of abstinence. Our results suggest that coping effectively with withdrawal symptoms can increase quitting success. Future research should identify how we can help smokers in a quitting program to develop effective coping strategies and thus increase their chances of staying abstinent.

Regarding physical exercise, the results showed that participants who never did any physical exercise were abstinent for a shorter period of time than participants who engaged in physical exercise moderately or frequently. Engaging in physical exercise frequently rather than only moderately resulted in no additional benefits. These results confirm that physical exercise can be an effective strategy to cope with withdrawal symptoms [23–25]. Finally, our study indicates that moderate exercise can be as effective as intensive exercise in prolonging abstinence.

Past attempts to quit and compliance to the pharmacological therapy had no significant influence on abstinence duration. In contrast to our results, past studies found that these factors were related to abstinence, motivation to change, and treatment adherence [15, 21]. We think that these differences may be due to the fact that our participants were all highly motivated to quit smoking. Future research can investigate this proposition by comparing participants high and low in motivation.

One limitation of our study is that all participants were employees of the University of Granada and had homogeneous socio-demographic characteristics (for example, all were employed, with high level of motivation). This makes it difficult to generalize our results to the general population. Further, the number of patients who used alcohol and/or tranquillizers in this study was rather small. However, an advantage of this study setting was that subjects attended all the follow-up visits, while usually smoking cessation studies are characterized by high lost-to-follow-up rates. This surprising result could be due to the fact that participants were in a controlled environment (Occupational Medicine Area, Prevention Service at the University of Granada). In particular, they were in continuous contact with the service regarding the smoking cessation program and regular health checks. This could have prevented any potential drop out.

In summary, the consumption of other drugs (alcohol and/or tranquilizers) shortened abstinence, while physical exercise, performance of treatment tasks, and coping with withdrawal symptoms prolonged abstinence. In particular, failure to perform the treatment tasks tripled the risk of relapse, while lack of coping doubled it. These results suggest that programs designed to help quitting smoking can benefit from the inclusion of these factors.
